# Measuring the Characteristic Topography of Brain Stiffness with Magnetic Resonance Elastography

**DOI:** 10.1371/journal.pone.0081668

**Published:** 2013-12-02

**Authors:** Matthew C. Murphy, John Huston, Clifford R. Jack, Kevin J. Glaser, Matthew L. Senjem, Jun Chen, Armando Manduca, Joel P. Felmlee, Richard L. Ehman

**Affiliations:** 1 Department of Radiology, Mayo Clinic College of Medicine, Rochester, Minnesota, United States of America; 2 Department of Physiology and Biomedical Engineering, Mayo Clinic College of Medicine, Rochester, Minnesota, United States of America; University College of London - Institute of Neurology, United Kingdom

## Abstract

**Purpose:**

To develop a reliable magnetic resonance elastography (MRE)-based method for measuring regional brain stiffness.

**Methods:**

First, simulation studies were used to demonstrate how stiffness measurements can be biased by changes in brain morphometry, such as those due to atrophy. Adaptive postprocessing methods were created that significantly reduce the spatial extent of edge artifacts and eliminate atrophy-related bias. Second, a pipeline for regional brain stiffness measurement was developed and evaluated for test-retest reliability in 10 healthy control subjects.

**Results:**

This technique indicates high test-retest repeatability with a typical coefficient of variation of less than 1% for global brain stiffness and less than 2% for the lobes of the brain and the cerebellum. Furthermore, this study reveals that the brain possesses a characteristic topography of mechanical properties, and also that lobar stiffness measurements tend to correlate with one another within an individual.

**Conclusion:**

The methods presented in this work are resistant to noise- and edge-related biases that are common in the field of brain MRE, demonstrate high test-retest reliability, and provide independent regional stiffness measurements. This pipeline will allow future investigations to measure changes to the brain’s mechanical properties and how they relate to the characteristic topographies that are typical of many neurologic diseases.

## Introduction

Magnetic resonance elastography (MRE) is a technique for performing noninvasive and quantitative “palpation” by measuring tissue stiffness [1]. MRE is a three-step process beginning with the introduction of shear waves into the tissue of interest via an external vibration source. A phase-contrast MRI pulse sequence with motion encoding gradients synchronized to the external vibration is used to image the resulting shear waves as they propagate through the tissue. Finally, an inversion algorithm is used to calculate a stiffness map (or elastogram) from the shear wave images.

Clinically, MRE is most often used to quantify liver disease severity from the early stages of fibrosis to cirrhosis [2]. More recently, a number of groups have begun to use MRE to study the mechanical properties of the brain and their potential to aid in the diagnosis of neurological diseases such as multiple sclerosis [3,4], normal pressure hydrocephalus [5], Alzheimer’s disease (AD) [6], and intracranial tumors [7,8]. Unfortunately these preliminary studies, while important to demonstrate the potential utility of stiffness as a novel biomarker of brain diseases, suffer from important technical limitations. 

The purpose of this work was to develop a new pipeline to address two significant limitations in the field, and to assess test-retest reliability of this technique in a cohort of 10 healthy control subjects. First, we used simulation experiments to demonstrate how atrophy can produce a systematic bias in MRE-based stiffness measurements. This issue is of critical importance when using MRE to study neurodegenerative diseases, where the disease group is expected to have smaller brain volumes on average compared to the control group. We then developed methods to remove this bias using novel, adaptive postprocessing techniques. Second, since diseases of the brain have characteristic topographies, it was critical to develop MRE as a tool to measure regional brain stiffness. Recent publications have presented regional brain property estimates [9,10]. We report methods to measure regional stiffness within the lobes of the brain with high test-retest reliability. Preliminary work has already demonstrated that stiffness changes in AD follow the known pathological topography of the disease [11]. These regional measurements are necessary to evaluate the specificity of brain stiffness changes, and also represent an initial step toward the use of brain stiffness for the differential diagnosis of diseases with varying topographies.

## Methods

### Simulation experiments

#### Simulated shear waves masked by spherical shells

To test postprocessing methods in a 3D object, we created simulated wave images (with no attenuation) masked by spherical shells of varying thickness. These images have the form u=sin(2πρ/λ-2πtf), where u is the displacement field, ρ is the distance from the point source, λ is the shear wavelength, t is time and f is the shear wave frequency. The shear waves were generated to simulate an object with a true stiffness of 3 kPa at 60 Hz with 3 mm isotropic image resolution. We simulated 3 motion encoding directions (in order to calculate the curl) by generating 3 wave images with the shear waves propagating from point sources of varying location outside the spherical shell masks. The wave images were masked with spherical shells of varying thicknesses, beginning with a 15 voxel thickness down to a 9 voxel thickness in increments of 2 voxels.

#### Finite element model (FEM) simulations

2D FEMs of brains simulating progressive atrophy were constructed in COMSOL Multiphysics (version 3.5.0.603, COMSOL AB, Stockholm, Sweden). An axial MR image from a normal subject was segmented to create a geometry consisting of brain tissue (not distinguishing gray and white matter) and cerebrospinal fluid (CSF). The FEM was a fluid-structure interaction model involving the plane strain mode of the Structural Mechanics Module (using a "mixed U-P" formulation for nearly incompressible materials) for the brain tissue and the pressure acoustics mode of the Acoustics Module for the CSF. Four geometries were constructed, one based on the original brain segmentation, and three others with progressive serial erosions of the brain geometry to represent atrophy. The mesh statistics for the four geometries are summarized in Table 1. Boundaries between the solid (brain) and fluid (CSF) domains were coupled in the solid domain using fluid-loading boundary conditions that applied an edge loading force based on the fluid acceleration, and in the fluid domain using structural acceleration boundary conditions that applied an acceleration to the fluid based on the acceleration of the solid. The outer edges of the brain geometry were prescribed a 10 micron vertical (anterior-posterior) displacement. The Young's modulus for the brain tissue was set to 8+3i kPa, the density was 1000 kg/m^3^, and the Poisson's ratio was 0.49995. The density of the CSF was set to 1000 kg/m^3^ with a speed of sound of 1540 m/s. The models were solved using a frequency-response analysis at 60 Hz using quadratic Lagrange elements and a direct solver (UMFPACK). The FEM solves for the in-plane (x and y) components of the motion in the tissue. The inversion algorithms described below are designed for 3D vector displacement data. To process the 2D FEM data, it was assumed that the z component of the motion was zero everywhere and that all derivatives in the z direction were zero.

**Table 1:** Mesh statistics for the 4 Finite Element Model geometries.

	**Geometry 1**	**Geometry 2**	**Geometry 3**	**Geometry 4**
Number of degrees of freedom	183,767	230,223	297,696	260,043
Number of mesh points	22,026	28,112	37,301	33,191
Number of triangular elements	42,741	53,827	71,607	63,631
Number of boundary elements	2,325	3,469	4,470	4,223
Number of vertex elements	587	881	998	952
Minimum element quality	0.237	0.757	0.763	0.342

#### Image processing: traditional MRE postprocessing methods

 A number of methods exist for calculating stiffness from the acquired wave images. The approach we use in the brain can be summarized by three steps. First, we calculate the first temporal harmonic of the vector curl of the acquired wave images. Applying the inversion algorithm to the first temporal harmonic ensures that the shear waves only contain displacements at the frequency of interest, while calculating the curl removes the effects of longitudinal waves that would otherwise produce artifacts in the inversion results [12]. For traditional postprocessing techniques, the partial derivatives for the curl calculation are estimated by central differences (i.e., convolution with a [-1, 0, 1] kernel in the direction of interest). Although the curl was not necessary for accurate inversion of the simulated shear waves, it was applied to maintain a consistent postprocessing point spread function between the simulation and *in vivo* data. Second, the curl images are smoothed to make the stiffness estimate more resistant to noise. We use a filter of the form (1-x^2^)^2^(1-y^2^)^2^(1-z^2^)^2^ where x, y and z are linearly spaced from -1 to 1 over the chosen window size [13]. Typically we use a 5x5x5 window, which is effectively a 3x3x3 filter since the filter will be 0 at the edges of the window. In the third and final step, three elastograms (one for each component of the curl) are calculated from the smoothed curl images using a direct inversion of the Helmholtz equation that is assumed to model the shear wave propagation [14]. These elastograms are combined into a single estimate using a weighted average based on the amplitude of shear wave motion in each of the components of the curl. Direct inversion calculates a complex shear modulus with the storage modulus as the real part and the loss modulus as the imaginary part. We convert the complex modulus to shear stiffness (the product of wave speed squared and density where density is assumed to be that of water) since shear stiffness is more resistant to noise and all three of these quantities (shear stiffness, storage modulus and loss modulus) are highly correlated with each other in these data. The stiffness is then calculated as the median value within the region of interest (ROI). Using this approach, stiffness in a given voxel is calculated using information from a 7x7x7 neighborhood (a 3x3x3 kernel for each of the curl calculation, smoothing and Laplacian calculation), which would be expected to introduce an edge artifact that is 3 voxels wide. For display purposes only, elastograms were smoothed with a 3x3x3 median filter, while quantitative measures were extracted from the unsmoothed elastograms.

#### Image processing: adaptive MRE postprocessing methods

 Adaptive MRE postprocessing follows the 3 major steps described above: 1) calculate the first temporal harmonic of the curl of the wave data; 2) smooth the curl images; and 3) calculate stiffness by direct inversion. However, after an ROI has been defined for analysis, adaptive methods use unique convolution kernels for edge voxels in steps 1 and 2 in order to reduce edge artifacts in the final elastogram. To calculate the curl, central difference estimates of the partial derivatives are used for any voxels whose convolution kernel lies completely within the ROI mask. For voxels that lie along the edge of the mask, derivatives are calculated using a nearest neighbor estimate. The smoothing filter can likewise be made adaptive by creating a unique filter for each voxel that sets any elements outside the mask to 0, and then normalizes the filter to the sum of its non-zero elements.

#### ROI selection

 To evaluate the impact of these postprocessing methods on edge-related bias, we measured stiffness in these simulations as a function of ROI size. The ROI began as a full mask of the object of interest, and then was serially eroded by 1 voxel from all edges up to 3 times. Erosion was performed with a 6-connected neighborhood for the 3D spherical shell simulations, and with a 4-connected neighborhood for the 2D FEM simulations.

### In vivo experiments

#### Subject recruitment

 This study was approved by the Mayo Clinic Institutional Review Board. All subjects were scanned after obtaining informed written consent. We scanned 10 volunteers (8 males and 2 females, ages 23 to 55) without known neurological disease 3 times each on one day to assess test-retest reliability. The subjects were removed from the scanner table, and the MRE apparatus was disassembled and reassembled between each MRE exam.

#### MRE data acquisition

 MRE data were collected using a modified single-shot, spin-echo EPI pulse sequence (SIGNA Excite, GE Healthcare, Waukesha, WI). Shear waves of 60 Hz were introduced using a pneumatic active driver (located outside of the scanner room) and a soft, pillow-like passive driver placed under the subject’s head as previously described [6]. The resulting motion was imaged with the following parameters: TR/TE=3600/62 ms; field of view (FOV)=24 cm; BW=±250 kHz; 72x72 imaging matrix reconstructed to 80x80; frequency encoding in the right-left direction; 3x ASSET (SENSE) acceleration; 48 contiguous 3 mm thick axial slices; one 4 G/cm, 18.2 ms, zeroth- and first-order moment nulled motion encoding gradient on each side of the refocusing RF pulse synchronized to the motion; motion encoding in the positive and negative x, y and z directions; and 8 phase offsets sampled over one period of the 60 Hz motion. The resulting images had 3 mm isotropic resolution and were acquired in less than 7 minutes. Two additional phase offsets with the motion turned off were collected for subsequent signal-to-noise ratio (SNR) calculations.

#### Image processing: wave image calculation

 For each of the x, y and z motion encoding directions, complex-valued phase difference images for each phase offset were calculated by taking the product of the complex-valued MR images with positive motion encoding and the complex conjugate of the images with negative motion encoding. These resulting images have a magnitude equal to the product of the magnitudes of the positive- and negative-encoded images, and phase equal to the difference of the phases of the positive- and negative-encoded images. To minimize slice-to-slice phase discontinuities, constant and slowly varying phase in the acquisition plane was removed by first filtering the complex-valued phase difference images with a 2D lowpass filter (3x3 rectangular window function in k-space) and then calculating the difference between the phase of the original complex-valued images and the phase of the low pass filtered images [15].

#### Image processing: brain mask and region assignment

 A brain mask and atlas regions for each subject were obtained using a separately acquired 3D IR-SPGR T1 weighted image (sagittal orientation; frequency encoding in the superior-inferior direction; TR/TE = 6.3/2.8 ms; flip angle = 11 degrees; TI = 400 ms; FOV = 27 cm; 256x256 acquisition matrix; BW = ±31.25 kHz; 1.75x ASSET acceleration in the anterior-posterior direction; and 200 1.2-mm slices). This image was segmented to calculate gray matter (GM), white matter (WM), and cerebrospinal fluid (CSF) content for each voxel as previously described [16]. A lobar atlas in a standard template space was warped to the subject’s T1 weighted image using a unified segmentation algorithm implemented in SPM5 [17]. The T1 weighted image was then registered to the magnitude data from the MRE exam (a T2 weighted image) with a 6 degree of freedom rigid body transformation, along with the segmentation images and the warped atlas. Finally, these images were resliced to calculate the GM, WM and CSF content (using trilinear interpolation), as well as the regional assignment of each voxel (using nearest neighbor interpolation) in MRE space. The brain mask was generated by marking any voxel where GM content plus WM content was greater than CSF content.

#### Image processing: regional stiffness calculation

The ROIs investigated included global (whole brain excluding cerebellum), frontal lobes, occipital lobes, parietal lobes, temporal lobes, deep GM/WM (insula, deep gray nuclei and white matter tracts) and the cerebellum. Each ROI was generated as the intersection between the brain mask and the warped atlas region. To calculate regional stiffness, the displacement data were first masked by the ROI for reasons which will be discussed later. Adaptive methods were used for calculating the curl and smoothing the masked data. As above, elastograms were calculated for each component of the curl using a direct inversion algorithm, which were then combined into one elastogram using a weighted average as above. Finally, the complex modulus was converted to shear stiffness and the stiffness was then summarized as the median over the ROI excluding 1 voxel from the edge of the ROI.

#### Image processing: correction of stiffness for noise-related bias

 In the final step of the pipeline, brain stiffness was corrected for potential bias due to noise. To begin this process, we calculated SNR maps (one for each component of the curl) as previously described [6]. Briefly, we calculated the amplitude of the first temporal harmonic of the curl images as the signal. We then calculated the curl of the motion-free data, which should be identically zero in every voxel if the data are noise-free. The noise level of each voxel was estimated by calculating the standard deviation of the motion-free curl images in sliding 3x3x3 windows excluding voxels outside the ROI mask. Finally, SNR was then calculated as the voxel-by-voxel ratio of these two quantities.

**Figure 1:**
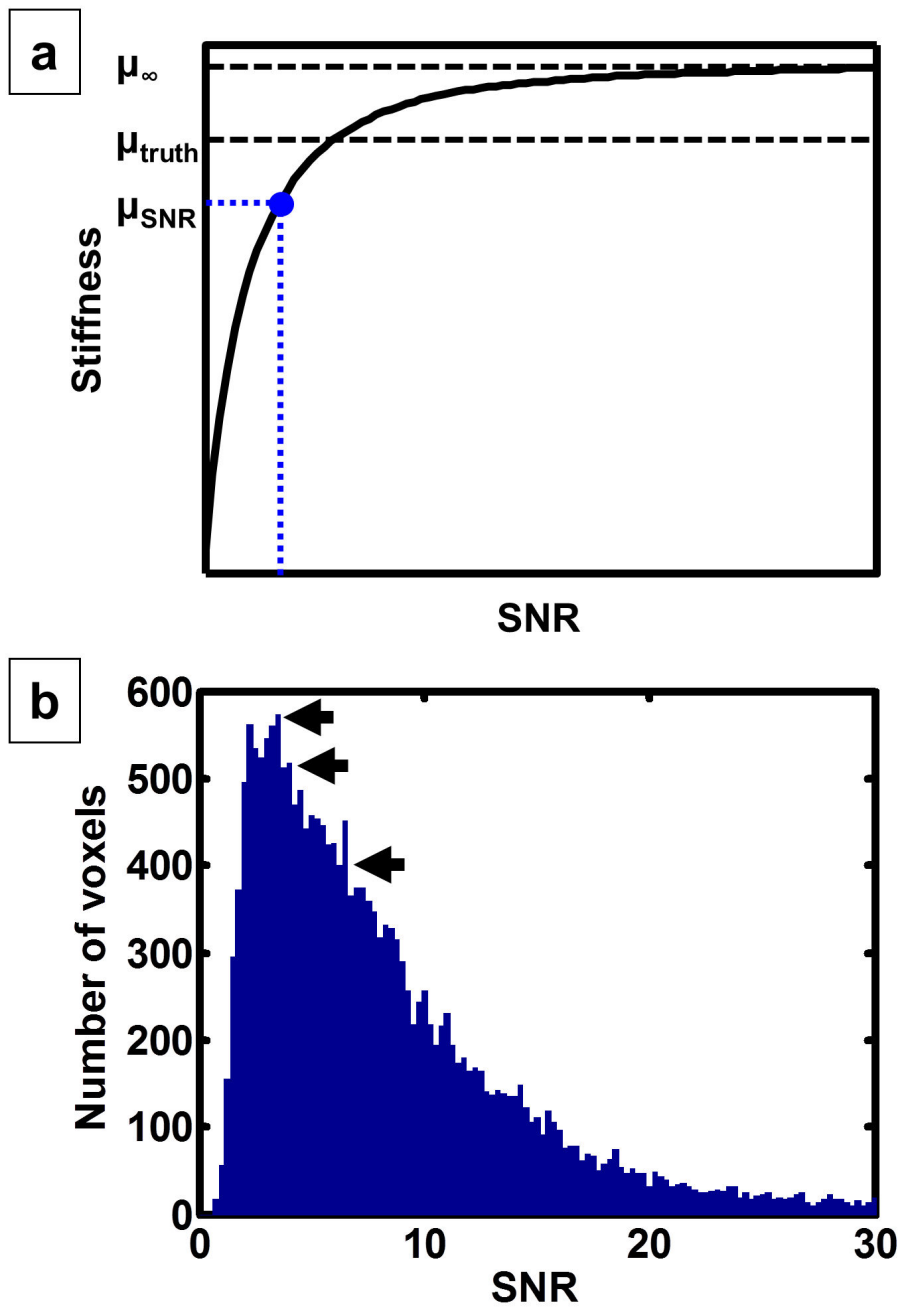
Relationship between stiffness and signal-to-noise ratio (SNR). a. In the noise-free case, measured stiffness will slightly overestimate the true stiffness due to discretization errors. The calculated stiffness then drops as noise increases. The SNR correction algorithm iteratively searches for the true stiffness that fits the measured stiffness and measured SNR. b. Example histogram of SNR within the brain. The distribution of SNR within the brain has a long right tail. Three summary measures of SNR were evaluated for the SNR correction algorithm: the mode (left-most arrow), the median of the most likely SNRs (middle arrow), and the median (right-most arrow).

 Based on simulation experiments, low SNR is known to lead to underestimated stiffness measurements using a direct inversion technique when calculating stiffness as the median over an ROI. We implemented an SNR correction algorithm that uses simulated data with known SNR to correct for this bias. As in the spherical shell-masked simulations above, these simulations have the form u=sin(2πρ/λ-2πtf), where u is the displacement field, ρ is the distance from the point source, λ is the shear wavelength, t is time and f is the shear wave frequency. The point source was placed outside the FOV, and the resolution and number of time offsets were set to mimic the *in vivo* data. Based on simulated data, the relationship between the true stiffness (μ_truth_), stiffness calculated from noise-free data (μ_∞_), and stiffness calculated at a particular SNR (μ_SNR_) is shown schematically in Figure 1a. A slight overestimate can exist in the noise-free stiffness estimate (μ_∞_) due to discretization errors [18]. Stiffness then systematically drops with increasing noise levels since noise has high spatial frequencies that are interpreted by the inversion algorithms as low wave speeds (i.e., soft tissue). The shape of this stiffness versus SNR relationship is dependent on the true stiffness, as softer materials have a flatter relationship while the stiffness measured in stiffer materials will decrease more quickly with decreasing SNR. The SNR correction algorithm presented here accounts for this stiffness dependency using an iterative approach to estimate the corrected stiffness given the measured stiffness (μ_m_) and the measured SNR. The approach is summarized as follows:

1. Initialize the stiffness of the simulation (μ_sim_) as the measured stiffness (μ_m_).
2. Calculate the simulated shear wave field with a wavelength corresponding to the simulated stiffness. This shear wave field has the same FOV and resolution as the *in* *vivo* data.
3. Apply the smoothing filter described above and calculate an elastogram using the direct inversion algorithm. Calculate the stiffness in the noise-free simulation (μ_∞_) by taking the median of this elastogram excluding 3 voxels from each edge.
4. Add noise to the simulated shear wave field by adding Gaussian noise with zero mean and a standard deviation equal to the inverse of the measured SNR.
5. Apply the smoothing filter and calculate an elastogram using the direct inversion algorithm. Calculate the stiffness in the noise-added simulation (μ_SNR_) by taking the median of this elastogram excluding 3 voxels from each edge.
6. Calculate a correction factor (CF) as the ratio of the stiffness calculated in the noise-free simulation to the stiffness calculated in the simulation with SNR equal to the measured SNR (CF= μ_∞_/μ_SNR_).
7. Calculate the difference between the measured stiffness multiplied by this correction factor and the stiffness calculated from the noise-free simulation (Δμ=CF*μ_m_ - μ_∞_), and increment the simulated stiffness μ_sim_ by Δμ.
8. Repeat steps 2 through 7 until Δμ is less than the tolerance (0.001 kPa for this work).
9. Report the corrected stiffness as the current simulated stiffness (μ_sim_).

This procedure converges quickly, typically requiring no more than 3 iterations. The difference between the uncorrected and corrected stiffness is typically on the order of hundredths of kPa.

#### Image processing: evaluation of SNR correction algorithm

The SNR correction algorithm was evaluated by testing the resistance of the stiffness measurements to additional artificial noise. Each MRE exam was reconstructed twice (once using the original data and once with additional noise). For the noise-added images, Gaussian noise with zero mean was added to the data in k-space prior to wave image calculation. The standard deviation of this noise was chosen to cut the median SNR in the global ROI to 50% of the original SNR. Stiffness in each region was then calculated using the original (μ_0_) and noise-added (μ_n_) data for each of the 30 exams (10 volunteers with 3 repeated exams), and the error in the inversion was calculated as (μ_0_- μ_n_)/μ_0_*100. Ideally the errors would be centered about 0 and have no relationship with the measured SNR. Therefore, the algorithm was tested by a paired Wilcoxon rank sum test between μ_0_ and μ_n_, and by a Spearman rank correlation between the inversion error and the measured SNR within each ROI.

#### Image processing: determination of regional SNR measure

The SNR correction algorithm above is applied on a global or regional basis, so the SNR in each ROI needs to be summarized by a single number (in actuality SNR varies spatially, due mostly to wave attenuation). SNR within the brain is not normally distributed, as seen in the example histogram in Figure 1b. Therefore, three summary statistics were examined, each was tested as the nominal SNR in the correction algorithm, and we chose the metric that minimized the average inversion error between original (μ_0_) and noise-added (μ_n_) data as defined above. The summary statistics tested include the mode (left-most arrow in Figure 1b), median (right-most arrow), and the median of the most likely SNRs (intermediate arrow). This final measure was computed by first finding the minimum number of bins that include at least half of all the voxels within the ROI, and then calculating the median SNR of the voxels that fell within the range of those bins. Since this metric was trained and tested in the same set of 30 exams, we cross-validated the SNR correction algorithm in a separate population (all imaging methods were the same as described above): 48 elderly subjects of age 72 to 89 including 32 cognitively normal controls, 8 subjects with mild cognitive impairment and 8 subjects with probable Alzheimer’s disease dementia.

#### Statistical analysis

 Test-retest repeatability in the 10 subjects was assessed by coefficient of variation (CV). To determine if stiffness in any region was different from any other region, we performed an ANOVA for repeated measures. Subsequent *post hoc* comparisons were made with paired t-tests. Correlations between regions were tested by Pearson correlation.

## Results

### Simulation experiments demonstrate edge-related bias due to atrophy that are eliminated using adaptive postprocessing methods

**Figure 2:**
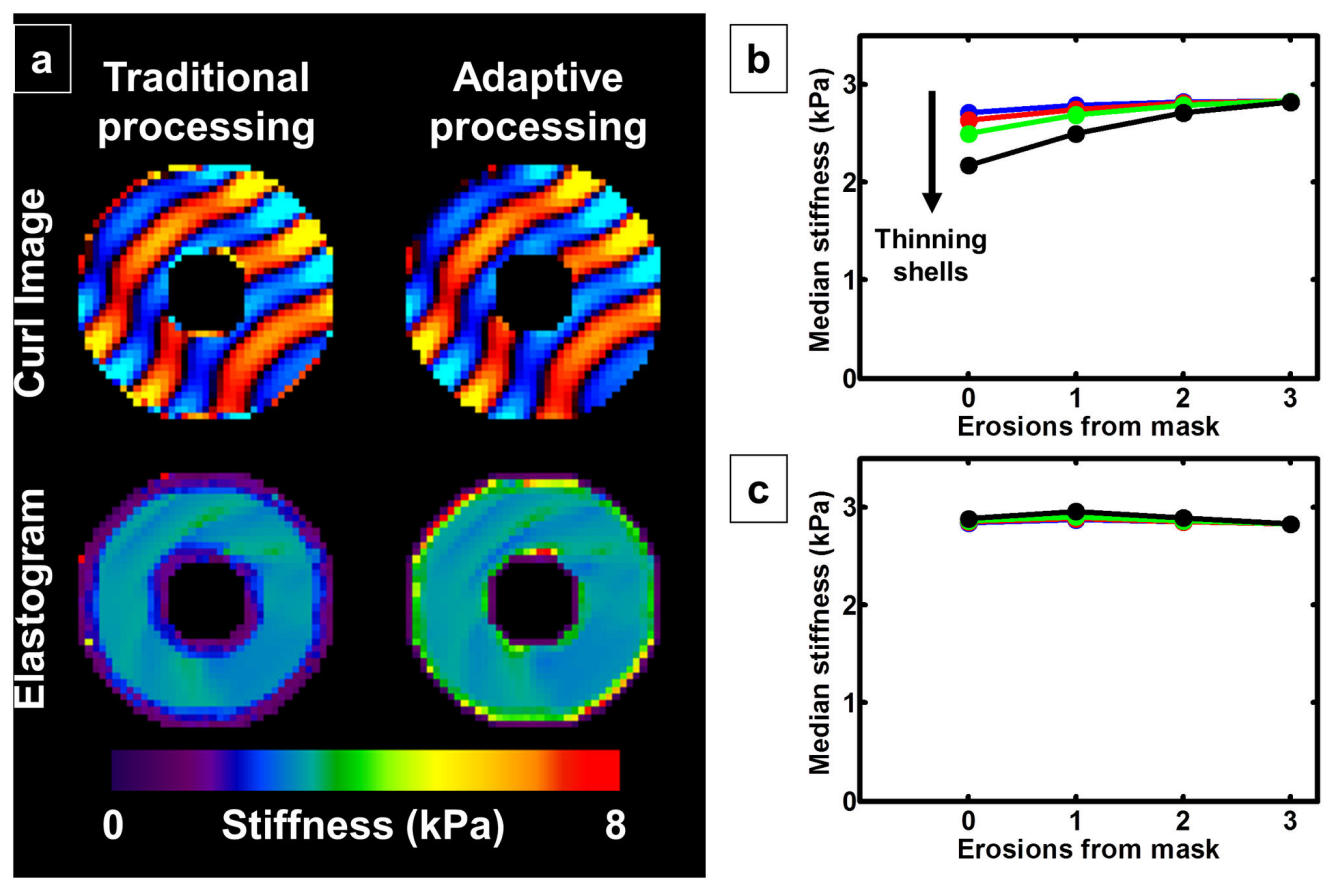
Spherical shell simulation. a. A curl wave image calculated with traditional postprocessing methods is shown in the top-left panel, while one calculated with adaptive methods is shown in the top-right. Note the edge discontinuities in the image on the left compared with that on the right. The elastogram calculated with traditional postprocessing in the bottom-left panel shows a larger edge-related bias (3 voxels wide) as compared to the elastogram calculated with adaptive processing in the bottom-right (1 voxel wide). b. Using traditional postprocessing methods, 3 erosions are required to completely remove edge-related bias. c. Using adaptive methods, no edge-related bias is apparent.

Example images of the spherical shell simulation are shown in Figure 2a. Note the edge discontinuities in the curl image calculated with traditional processing (top-left). On the other hand, curl images calculated with adaptive methods (top-right) are significantly smoother right up to the edge of the object. In the stiffness map calculated with traditional processing (bottom-left), regions within 3 voxels of an edge give an underestimated stiffness. However when using adaptive methods (bottom-right), only regions within 1 voxel of an edge give an underestimated stiffness. As the shells decrease in thickness, the ratio of the number of edge-artifacted voxels to the total number of voxels increases. As a result, if the entire object is used as the ROI, then the calculated stiffness will continue to decrease as the shells become thinner as shown in Figure 2b. Figure 2b shows that when using traditional postprocessing techniques, 3 erosions of the ROI are necessary to eliminate the bias. The same plot is shown when using adaptive methods in Figure 2c, indicating no edge-related bias.

**Figure 3:**
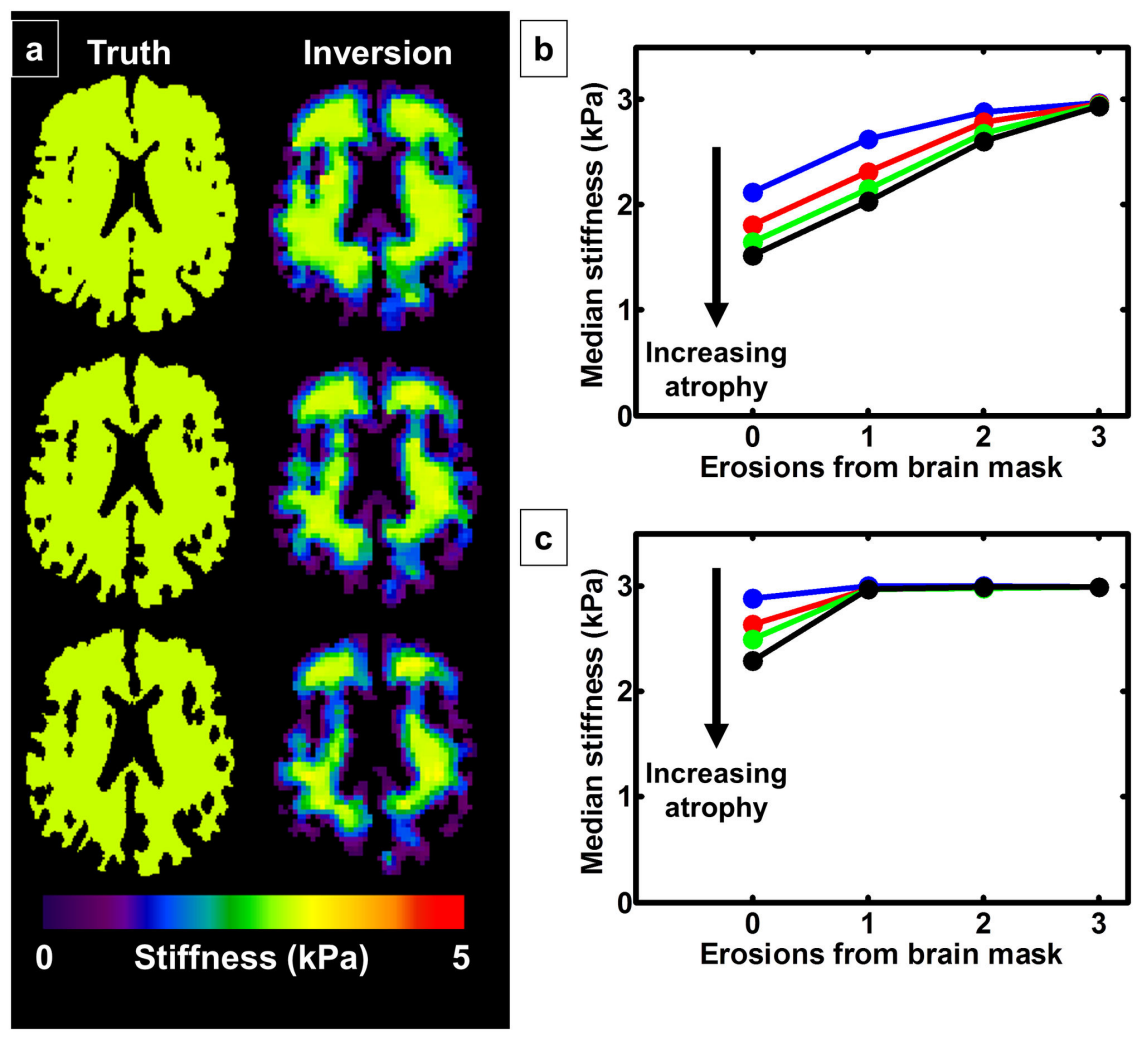
Finite element model (FEM) simulations. a. The true stiffness maps given to the FEM are shown on the left with increasing atrophy from top to bottom. The corresponding elastograms are shown on the right after downsampling the wave images to 3 mm resolution, calculating the curl, smoothing and calculating stiffness with a direct inversion algorithm. Voxels near the edge of the brain provide underestimates of the true stiffness. Increasing atrophy causes a systematic bias toward underestimated stiffness. b. Using traditional postprocessing methods, this bias can only be completely removed by eroding the ROI by 3 voxels from every edge. c. Using adaptive postprocessing methods, the edge-related bias is removed after only 1 erosion of the ROI.

This edge-related bias was also evaluated in the FEM simulations to test the adaptive methods in a more realistic geometry. The left column of Figure 3a shows the true stiffness maps given to the FEM with increasing simulated atrophy from the top row to the bottom row. In the right column are the corresponding elastograms after downsampling the FEM wave images to 3 mm resolution, calculating the curl, smoothing and performing a direct inversion, as done for a brain MRE exam. Note how the voxels near the edge of the brain indicate stiffness below that of the true stiffness, and as atrophy increases, the ratio of these edge voxels with artifact to the total number of voxels increases. As above, if the entire brain mask is used as the ROI, then the calculated stiffness will systematically decrease with increasing atrophy as shown in Figure 3b. Just as in the spherical shell simulations, this bias can only be completely removed by eroding the ROI by 3 voxels from every edge of the brain when using traditional postprocessing techniques. On the other hand, this edge-related bias is reduced when using adaptive methods as shown in Figure 3c. As expected, only 1 erosion from the brain mask is necessary to remove the edge-related bias.

### Evaluation of SNR correction algorithm

**Figure 4:**
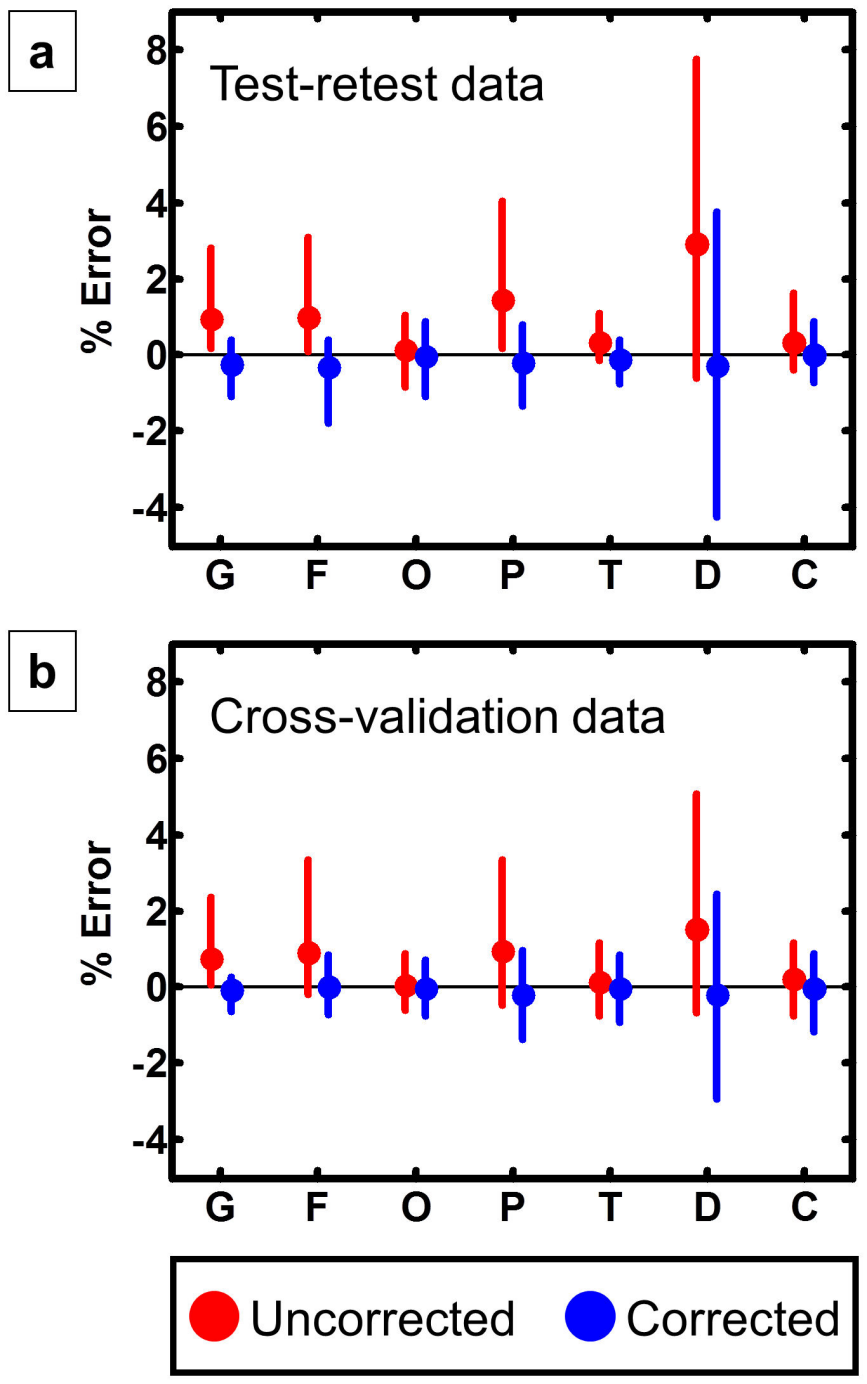
Effects of the SNR correction algorithm on noise-related bias. a. Errors for the test-retest data are shown in the top panel. The markers represent the average inversion error over all exams before (red) and after (blue) SNR correction, and the bars represent the range from percentile 2.5 through 97.5 for each region (F=frontal lobes, O=occipital lobes, P=parietal lobes, T=temporal lobes, D=deep GM/WM, C=cerebellum). Two ROIs had a significant difference between μ_0_ and μ_n_, as well as a significant relationship between inversion error and SNR (global and frontal). The parietal lobe ROI had only a significant relationship between inversion error and SNR. The absolute value of the errors was decreased by the algorithm in every ROI and the average error was never larger than 0.29%. b. For cross-validation, errors in the elderly cohort are shown in the bottom panel. The parietal lobe ROI had a significant relationship between inversion error and SNR, but no ROI had significant differences between μ_0_ and μ_n_ in this sample.

 Error was minimized by using the median of the most likely SNRs as the summary metric. Using the median to summarize SNR led to under-corrected stiffness values (i.e., positive inversion errors), and using the mode resulted in over-corrected stiffness values (i.e., negative inversion errors). The inversion error as a function of region both with and without SNR correction is shown in Figure 4a. The markers represent the average error over the 30 scans (10 subjects, 3 repeated scans), and the bars represent the range from a percentile of 2.5 to 97.5. In this data set, two ROIs had a significant difference between stiffness calculated with the original data (μ_0_) and stiffness calculated with the noise-added data (μ_n_), as well as a significant relationship between inversion error and SNR (global and frontal lobes). One more ROI had only a significant relationship between inversion error and SNR (parietal lobes). However, these errors are small in absolute terms as the largest average inversion error is 0.29%, or equivalently in units of stiffness, 0.0093 kPa.

 The SNR correction algorithm was also cross-validated in an elderly cohort, which is summarized in Figure 4b. As above, the error is decreased in every ROI by applying the SNR correction algorithm. In this group, only one ROI had a significant relationship between inversion error and SNR (parietal lobes). No ROI demonstrated a significant difference between μ_0_ and μ_n_.

### Regional brain stiffness measurement

**Figure 5:**
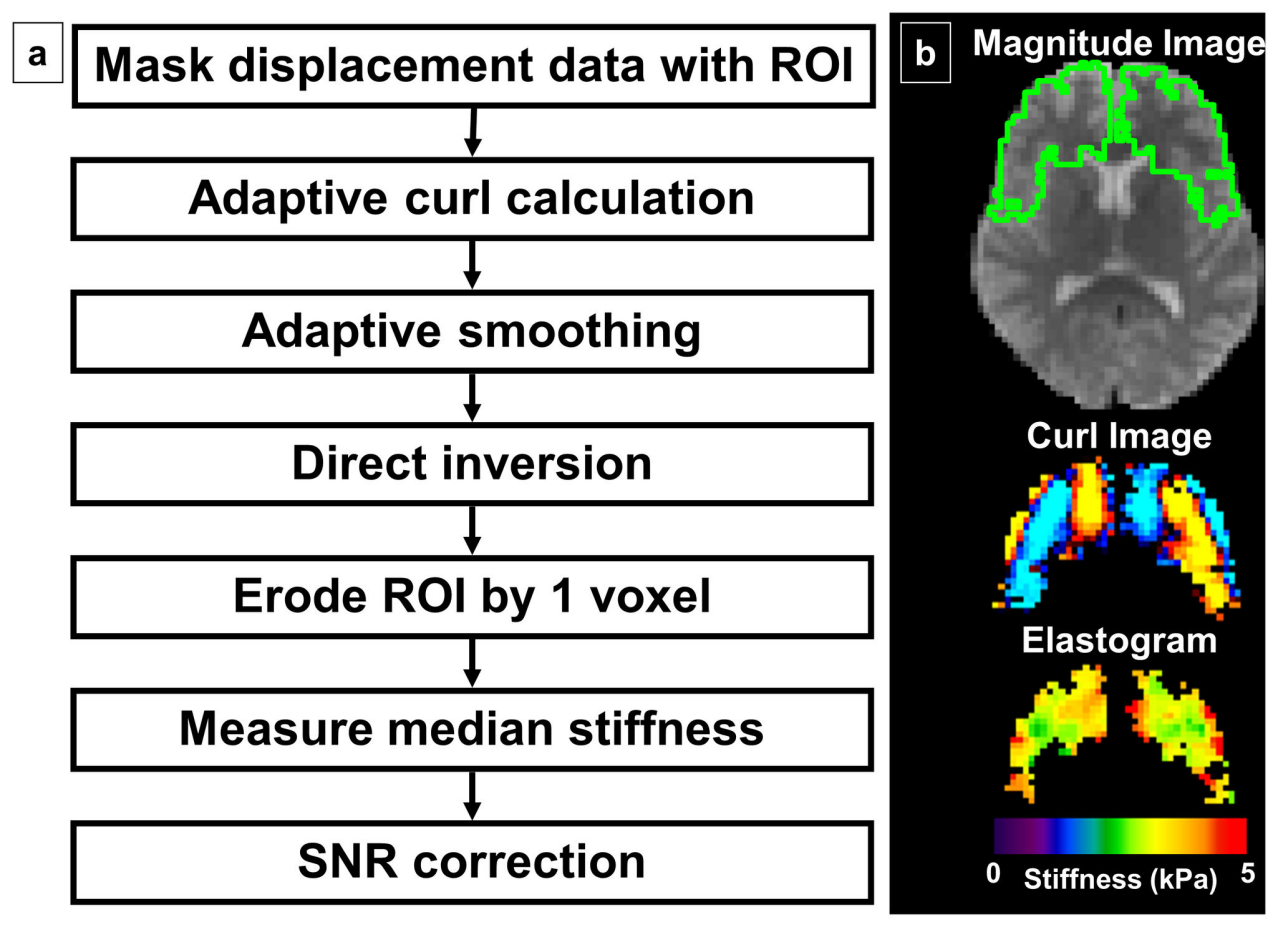
Summary of the regional MRE pipeline. a. The steps for regional stiffness measurement are summarized in the left panel. b. Example images are shown in right panel. At the top is the magnitude image from the MRE data with the frontal lobe ROI outlined in green. Below that image is the ROI-specific wave image, followed by the ROI-specific elastogram.

Based on the above results we developed a pipeline to measure regional brain stiffness, which is summarized in Figure 5a. In our initial attempts to measure regional stiffness, the full displacement field was used to calculate a single elastogram that was then parceled into different ROIs to measure regional stiffness. The downside to this approach is that the elastogram is effectively a lowpass filtered image of the true underlying stiffness, meaning the stiffness in a given region will impact the stiffness calculated in any adjacent region. Therefore, in the pipeline we have implemented, the wave images are masked by the ROI as the very first step, and unique curl images and elastograms are calculated for each ROI. In this way, stiffness in a particular lobe is not only resistant to partial volume effects from outside the brain but also from neighboring regions of the brain. This process is illustrated in Figure 5b. On top is an example MRE magnitude image with the frontal lobe ROI outlined in green. Below that is the ROI-specific curl image, followed by the ROI-specific stiffness map. Note that this pipeline cannot be implemented in all cohorts using non-adaptive processing methods, as 3 erosions from an atrophied brain mask will leave no voxels in some ROIs. The edge-adaptive processing is thus a key step in achieving highly reproducible regional stiffness estimates.

**Figure 6:**
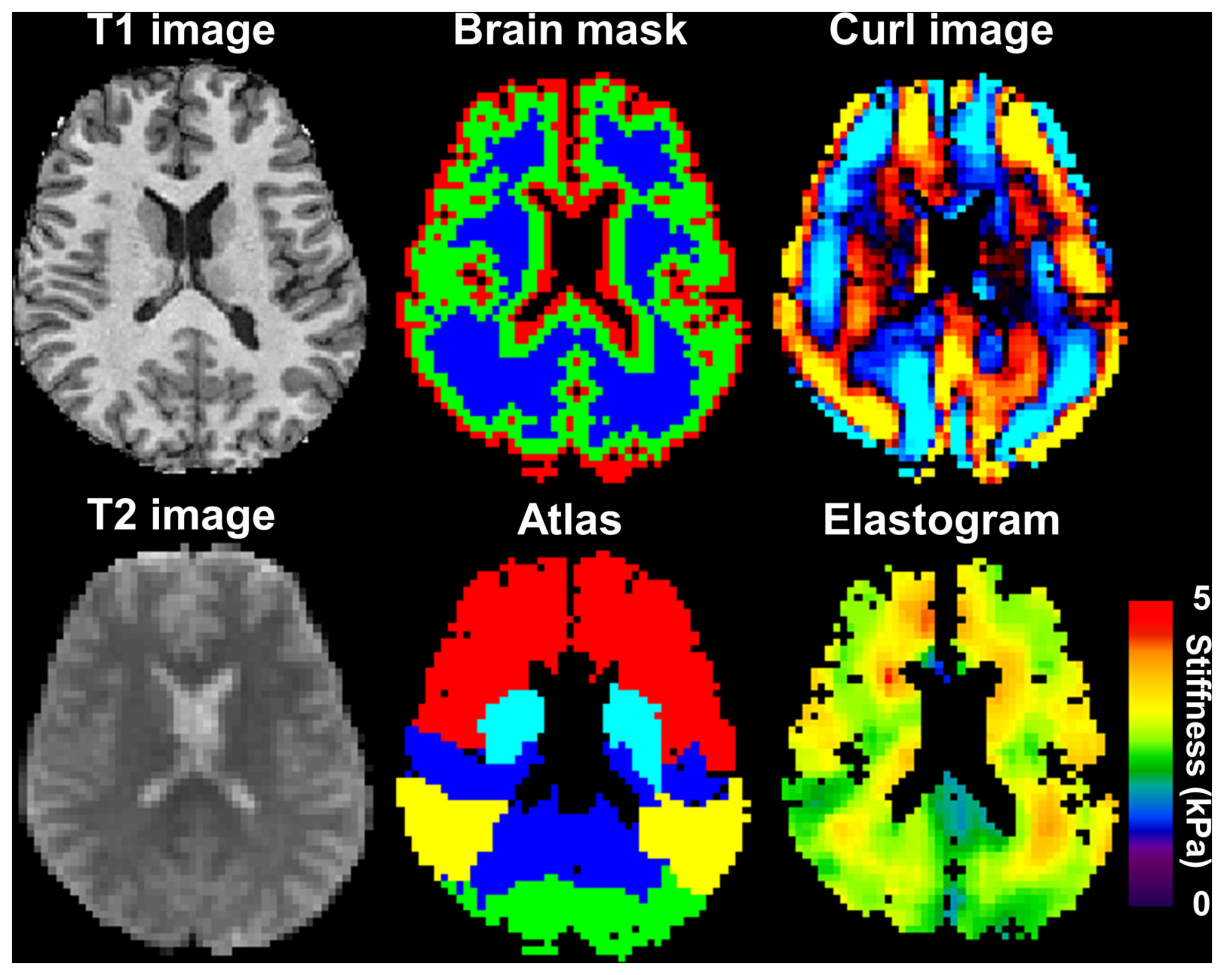
Example images of the global ROI. A T1 weighted image is shown in the top-left, and the corresponding T2 weighted magnitude image from the MRE data is shown in the bottom-left. In the top-center panel is the full brain mask in red, the brain mask after 1 erosion in green and the brain mask after 3 erosions in blue. The difference between the green and blue masks indicates the number of voxels saved by using adaptive methods while incurring no edge-related bias. In the bottom-center panel is the warped atlas in MRE space (red=frontal lobes, green=occipital lobes, blue=parietal lobes, yellow=temporal lobes, cyan=deep GM/WM, the global ROI is the union of all ROIs except for the cerebellum). The wave image is shown in the top-right panel, and the resulting elastogram after 1 erosion is in the bottom-right panel.

Example images for the global ROI are shown in Figure 6. A T1 weighted image is shown in the top-left panel, while the corresponding T2 weighted MRE magnitude image is shown in the bottom-left. The top-center image shows the brain mask generated from the T1 segmentations. The red voxels indicate the full brain mask. Green voxels show the brain mask after 1 erosion and blue voxels show the brain mask after 3 erosions, indicating how many voxels are saved by using adaptive postprocessing methods. The regional assignment of each voxel is shown in the bottom-center panel (red=frontal lobes, green=occipital lobes, blue=parietal lobes, yellow=temporal lobes, cyan=deep GM/WM). The curl wave image is shown in the top-right panel, and the elastogram after 1 erosion is shown in the bottom-right.

**Figure 7:**
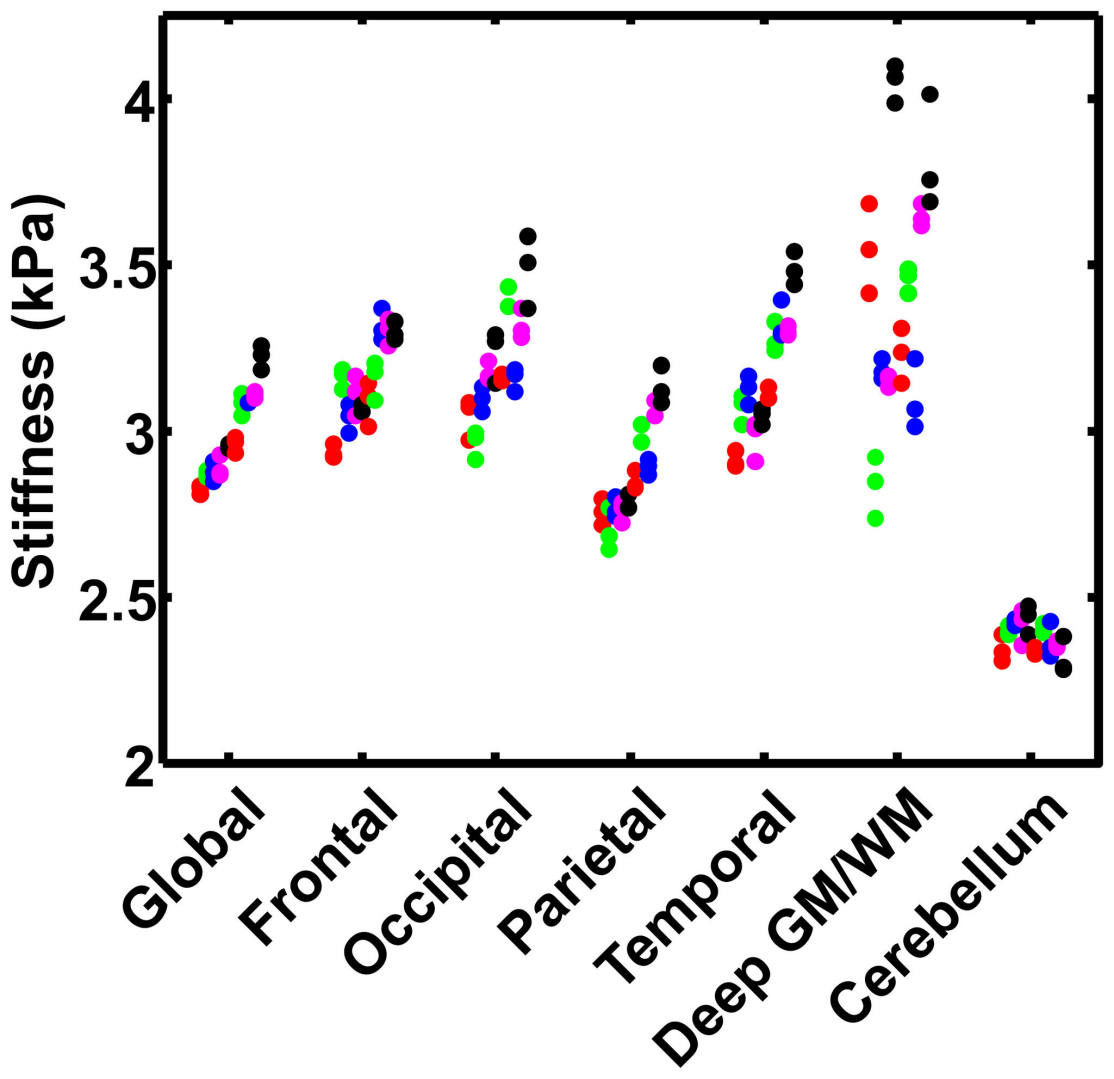
Summary plot of the repeatability of regional brain stiffness measurements. Each region contains 10 columns (corresponding to the 10 volunteers sorted by average global stiffness) and each column contains 3 markers (corresponding to the 3 MRE exams). The results indicate high test-retest repeatability (summarized in Table 2).

The repeatability of regional brain stiffness measurements is summarized in Figure 7. Each region contains 10 columns (corresponding to the 10 volunteers sorted by average global stiffness), and each column contains 3 markers (corresponding to the 3 MRE exams). The results indicate that the technique is highly repeatable for measuring global brain stiffness with median and maximum CVs of 0.67% and 1.11%, respectively. The lobes of the brain, deep GM/WM and cerebellum regions also indicate strong test-retest reliability with a median CV no greater than 1.98% and a maximum CV no greater than 4.48%. Table 2 summarizes the median stiffness, median ROI size (in number of voxels), median CV and maximum CV for each region. Since the subjects in this study were young volunteers, we were also able to assess repeatability of regional brain stiffness using traditional processing methods (requiring 3 erosions from the ROI to eliminate edge-related bias). The average CV over the 10 subjects and the 7 ROIs was 1.35% when using adaptive methods and 2.48% when using traditional methods (p=1.8e-8, paired t-test).

**Table 2:** Summary of repeatability data by region.

**Regional**	**Median stiffness (kPa)**	**Median ROI size (number of voxels)**	**Median CV (%)**	**Maximum CV (%)**
Global	2.99	27,153	0.67	1.11
Frontal lobes	3.15	8,413	1.27	2.18
Occipital lobes	3.21	2,474	1.25	3.08
Parietal lobes	2.87	4,313	1.11	2.44
Temporal lobes	3.17	4,914	1.41	2.03
Deep GM/WM	3.41	1,424	1.99	4.48
Cerebellum	2.38	3,431	1.14	2.33

We performed ANOVA for repeated measures, which indicated significant differences both between individuals (p<0.01) and between brain regions (p<0.001). *Post hoc* comparisons indicated that stiffness in the parietal lobes and cerebellum were both significantly different from all other regions. In addition, frontal lobe stiffness versus deep GM/WM stiffness met trend level significance (p<0.1). We also calculated a correlation matrix to demonstrate the relationship of stiffness between regions of the brain within an individual. The results indicate that stiffness within the four lobes of the brain track together (5 out of 6 correlations are significant at the p<0.05 level), but stiffness within the deep GM/WM and the cerebellum are independent of the rest of the brain (1 out of 9 correlations significant at the p<0.05 level).

## Discussion

In this work, we presented novel MRE postprocessing methods to measure regional brain stiffness. These techniques are resistant to noise- and edge-related biases that are common in the field, and reveal new insights into the topographical distribution of the mechanical properties of the brain in healthy control subjects.

The simulation data in this work provide a causal link between brain geometry and biased stiffness measurements when the ROI does not properly account for edge artifacts in the elastogram. To fairly compare brain stiffness between subjects in varying disease states, the processing methods used must be resistant to changes in geometry such as those presented in this work. As mentioned earlier, this bias due to brain geometry is particularly important when investigating diseases of the brain that are known to cause changes in brain morphometry (such as brain atrophy due to neurodegenerative disease). A correlation between brain volume and MRE-based stiffness measurements has been previously reported [3,19], and without methods that are resistant to changes in geometry it cannot be known whether that correlation is a reflection of biologically driven changes in brain stiffness, edge artifacts in the postprocessing, or likely some combination of both factors. Furthermore, ROI selection must consider the resolution limits of MRE. Measuring the stiffness within small structures will not provide an accurate stiffness estimate without significantly improved image resolution or further advancements in postprocessing techniques to correct for edge artifacts.

With these findings in mind, we developed a novel MRE pipeline to measure regional stiffness within the brain. This pipeline uses adaptive processing methods to significantly reduce the edge artifacts discussed above, thus saving most edge voxels. Retaining as many edge voxels as possible may be of critical importance for detecting biological signals. For example, amyloid is deposited in the cortex in Alzheimer’s disease and white matter injury occurs most frequently in the periventricular regions, both of which are along the surface of the brain. 

**Figure 8:**
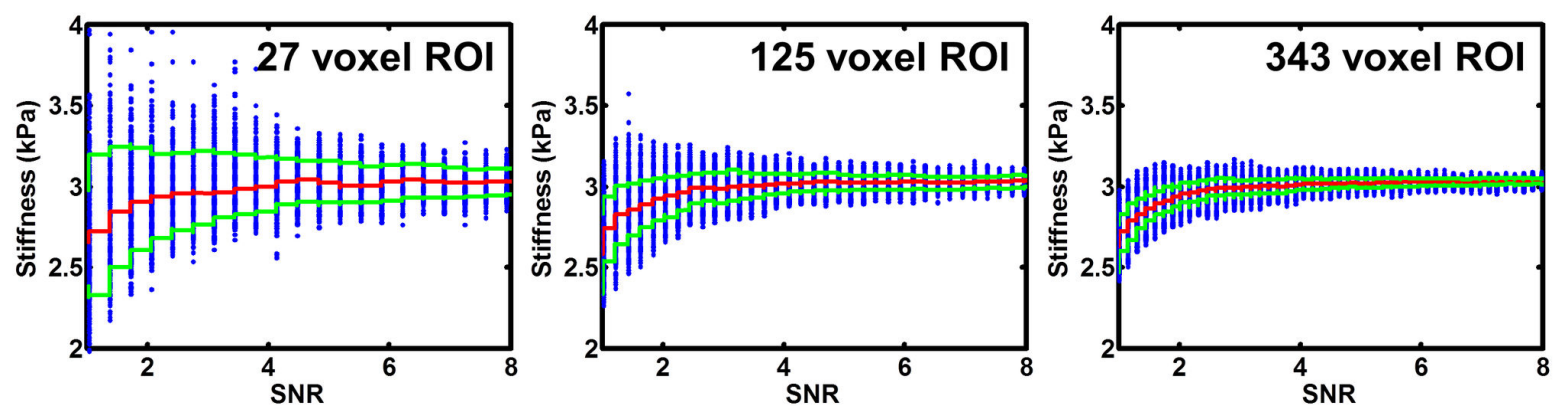
Median stiffness as a function of SNR and ROI size. As the ROI size increases, the median stiffness remains constant but the precision of the stiffness measurement improves.

These developed adaptive methods also improved the reliability of regional brain stiffness measurement. This finding can be explained by two factors. First, due to wave attenuation, shear wave amplitude is strongest at the outer edge of the brain and decreases as the waves propagate toward the center the brain. Therefore, the average SNR within any ROI will be increased by retaining these voxels near the edge that possess the largest shear wave amplitude. Second, increasing the number of samples in an ROI will improve test-retest reliability. This concept is demonstrated in simulation results that are summarized in Figure 8. In this simulation (true stiffness of 3 kPa at 60 Hz), the SNR is spatially varied and then summarized by the median in sliding windows (3x3x3, 5x5x5 or 7x7x7). Each blue marker represents the median stiffness in one of these sliding windows versus its median SNR. The red lines represent the median value of those markers as a function of SNR, while the green lines represent the 10^th^ and 90^th^ percentiles. Note that the median stiffness does not change with increasing ROI size (meaning there is no systematic bias with ROI size), but the range of the stiffness measurements decreases with increasing ROI size (meaning the precision of stiffness measurement improves as the number of voxels in the ROI increases).

In this work, we found a median global brain stiffness of 2.99 kPa at 60 Hz (range: 2.83 to 3.23 kPa) in young to middle aged normal volunteers, which is in close agreement with the most recent brain MRE literature. Johnson et al. scanned 3 young volunteers to evaluate their newly developed mulitshot, spiral EPI pulse sequence and reported an average shear modulus of 2.43+1.21i kPa in white matter at 50 Hz [20]. This complex modulus corresponds to a shear stiffness of 2.86 kPa [14]. Streitberger et al. performed a multifrequency MRE study of brain viscoelasticity using a two-parameter springpot model to study chronic-progressive multiple sclerosis. In the younger of their two control groups (most comparable to our subjects), they reported a shear modulus of 3.545 kPa and an α value of 0.2928, which is a measure of how viscous or elastic the tissue may be [3]. Evaluating their model at 60 Hz indicates a shear modulus of 2.42+1.20i kPa, or a shear stiffness of 2.85 kPa. Finally, Zhang et al. measured the shear modulus in both the cerebrum and the cerebellum at 80 Hz in 8 subjects [21]. In the cerebral WM they reported an average shear modulus of 2.41+1.21i kPa (shear stiffness of 2.85 kPa), and in cerebellar WM they reported a modulus of 1.85+1.10i kPa (shear stiffness of 2.31 kPa). Relative to global stiffness, we likewise observed lower stiffness in the cerebellum (median: 2.38 kPa, range: 2.32 to 2.44 kPa). We should note that the works outlined above assume an isotropic material, which may introduce errors particularly in the anisotropic white matter. These errors may be reduced by using an anisotropic model to solve for brain stiffness such as the method introduced by Romano et al. [22]. Furthermore, the inversion used in this work assumes linear elastic behavior. While the mechanical properties of the brain are non-linear, the displacements and strains generated by this method are so small (maximum shear displacements on the order of tens of microns and maximum shear strains on the order of tenths of a percent) that the effect of non-linear behavior on the stiffness calculation is expected to be negligible.

This work has provided new insights into the topographical distribution of stiffness within the brain. Based on these results, we observed that on average (considering the 4 lobes of the brain), stiffness is greatest in the occipital lobes, followed by temporal, then frontal (though stiffness was similar in these 3 regions) and finally the parietal lobes. Whether lobar variation in stiffness is explained by fundamental brain architectural differences at the lobar level is the subject of future investigation. Perhaps more importantly, these methods will allow us to investigate disease-related changes in brain stiffness on a regional basis to demonstrate that brain stiffness is not only sensitive to biological processes but also offers specificity (i.e., brain stiffness only changes in regions of the brain known to be impacted by the disease), as has been indicated by preliminary work on Alzheimer’s disease [11].

In conclusion, we used two types of simulations to demonstrate how atrophy can bias MRE-based stiffness measurements toward an underestimate of the true stiffness. We then developed adaptive techniques to reduce these edge artifacts. Using the simulations to test these methods, we demonstrated that the edge-related bias can be eliminated by eroding the ROI by only 1 voxel from the brain’s surface. Finally, we used these methods to develop a pipeline to measure regional brain stiffness in 10 healthy control subjects. These results indicate high test-retest reliability, and also that brain stiffness follows a characteristic topography.
